# The Long Non-coding RNA lnc-DMP1 Regulates *Dmp1* Expression Through H3K27Ac Modification

**DOI:** 10.3389/fgene.2020.00233

**Published:** 2020-03-18

**Authors:** Xin Xia, Yi Ruan, Boya Li, Yansong Yu, Xiangbo Kong, Peilin Zhuang, Hong Wu

**Affiliations:** Department of Stomatology, Sun Yat-sen Memorial Hospital, Sun Yat-sen University, Guangzhou, China

**Keywords:** long non-coding RNA, Dentin matrix protein 1, transcriptional regulation, mice mandible, MC3T3-E1 cell, H3K27Ac

## Abstract

Several long non-coding RNAs (lncRNAs) have been reported regulate the expression of neighbor protein-coding genes at post-transcriptional, transcriptional and epigenetic levels. *Dmp1* (*Dentin matrix protein 1*), encoding a non-collagenous extracellular matrix protein, plays an important role in dentin and bone mineralization. However, the transcriptional regulation of lncRNA on *Dmp1* has not been reported. In this study, we identified a novel lncRNA named lnc-DMP1, which is near the *Dmp1* gene region and undergoes remarkable changes during mandible development. lnc-DMP1 is co-localized and significantly expressed correlation with *Dmp1* in embryonic and postnatal mouse mandibles. In MC3T3-E1 cells, lnc-DMP1 positively regulates DMP1 expression and skeletal mineralization. Furthermore, lnc-DMP1 induces the promoter activity of *Dmp1* by modulating H3K27Ac enrichment in the *Dmp1* promoter. In conclusion, our results indicate that lnc-DMP1 is a novel lncRNA near the *Dmp1* gene region and regulates *Dmp1* expression by modulating the H3K27 acetylation level of *Dmp1* promoter.

## Introduction

Long non-coding RNAs (lncRNAs) are defined as a subgroup of non-coding RNA molecules that consist of at least 200 nucleotides and exhibit no or limited protein-coding capability (Roberts et al., 2014; Marchese et al., 2017). Since the major role of *Xis*t in X-chromosome inactivation was first described (Brockdorff et al., 1992; Brown et al., 1992), studies have demonstrated that lncRNAs function in multiple cellular processes, such as genomic locus imprinting (Kanduri, 2016), antiviral response (Fortes and Morris, 2016) and differentiation and development (Fatica and Bozzoni, 2014). Studies have also investigated various mechanisms underlying lncRNA functions. Some nuclear lncRNAs are expressed from imprinted loci function as molecular scaffolds that recruit chromatin-modifying complexes and regulate gene expression in *cis* by altering the chromatin structures of target genes (Lee and Bartolomei, 2013; Melo et al., 2013). Other lncRNAs modulate gene expression in *trans* by interfering with transcriptional machineries or maintaining the structures of nuclear speckles (Prasanth et al., 2005; Clemson et al., 2009; Sunwoo et al., 2009). Some cytosolic lncRNAs have been suggested to regulate mRNA splicing, mRNA decay, protein translation and protein stability (Yoon et al., 2013; Quinn and Chang, 2016).

Dentin matrix protein 1 (DMP1), a highly phosphorylated acidic non-collagenous phosphoprotein, is initially identified by cDNA cloning in rat odontoblasts and highly expressed in bone tissues (George et al., 1993; D’Souza et al., 1997; Hirst et al., 1997; MacDougall et al., 1997; Feng et al., 1998). DMP1 plays a key role in the control of mineralization and phosphate homeostasis in dentin and bone (Qin et al., 2007). The deletion of *Dmp1* leads to severe defects in the odontogenesis of the dentin and cartilage formation of bones in mice (Ye et al., 2004, 2005). Importantly, the loss of *Dmp1* results in autosomal recessive hypophosphatemic rickets, a novel disorder in humans (Feng et al., 2006; Lorenz-Depiereux et al., 2006).

Narayanan et al. (2003) investigated the transcriptional regulation of *Dmp1* by c-Fos and c-Jun, two AP-1 transcriptional factor family members, which play important roles in early osteoblast differentiation. They also demonstrated that JunB can interact with p300 and dramatically modulate the promoter activity of *Dmp1* during osteoblast mineralization (Narayanan et al., 2002). The transcription factor TCF11 binds to the *Dmp1* promoter and activates *Dmp1* transcription in osteoblasts (Jacob et al., 2014). However, the effect of lncRNA on the transcriptional regulation of *Dmp1* has yet to be reported.

Since the transcription factor JunB regulates the expression of *Dmp1* gene through interaction with the transcriptional coactivator p300, which is a histone acetyltransferase (Narayanan et al., 2002). Meanwhile, lncRNAs could function as molecular scaffolds of histone-modifying enzyme to modulate the expression of target gene through specific histone modification such as methylation and acetylation (Marchese et al., 2017). So, we assumed that lncRNAs could play a certain regulatory role in the expression of *Dmp1*. Thus, we used lncRNA-seq technology to screen lncRNAs near the promoter of *Dmp1* and differently expressed between embryonic and postnatal mouse mandibles. lnc-DMP1 was selected on the basis of the lncRNA sequence profiles. Then, the expression patterns of lnc-DMP1 and *Dmp1* in the embryonic and postnatal mouse mandibles were detected through qRT-PCR and RNA-fluorescence *in situ* hybridization (FISH) to investigate whether lnc-DMP1 was significantly correlated with *Dmp1*. Subsequently, whether lnc-DMP1 could participate both mRNA and protein expression of DMP1 and mineralization were tested in osteoblast cells MC3T3-E1. Finally, luciferase assay and chromatin immunoprecipitation (ChIP) methods were performed to investigate whether lnc-DMP1 could transcriptionally regulate the *Dmp1* expression in MC3T3-E1 cells by modulating the enrichment of H3K27Ac in *Dmp1* promoter.

## Materials and Methods

### Animals and Cell Lines

Embryonic (16, 18, and 20 days old) and postnatal (2 and 4 weeks old) C57 mice were purchased from the Animal Experiment Center of Sun Yat-sen University (Guangzhou, China). The body weight of the mice was 10–15 g, and the mice were fed at room temperature (20–22°C) (License code: SYKX: 2012-0081). The mice were anesthetized by intraperitoneally injecting 5% chloral hydrate (0.1 mL/10 g) and then fixed on a dry ice anatomy bench. The mandibles were extracted under a microscope, frozen in liquid nitrogen and stored at −80°C. After sampling was completed, the mice were sacrificed by dislocating their necks and then treated in accordance with the death treatment method for experimental animals. All animal experiments were conducted following the Ministry of Health national guidelines for housing and care of laboratory animals and performed in accordance with institutional regulations after review and approval by the Institutional Animal Care and Use Committee at the Sun Yat-sen University (IACUC-DB-13-132).

The MC3T3-E1 cell line (American Type Culture Collection, Manassas, VA, United States) was cultured in α-MEM (Gibco, New York, NY, United States) and pretreated with 10% fetal bovine serum. The cells were cultured in an incubator with humidified atmosphere and 5% CO_2_ at 37°C.

### lncRNA Sequencing Assay

Total RNA was isolated from the mandibles of embryonic (18 days old) and postnatal (2 weeks old) C57 mice with TRIzol reagent (Thermo Fisher Scientific, Carlsbad, CA, United States) in accordance with the protocol. RNA quantity and quality were evaluated with Agilent Bioanalyzer 2100. The qualified RNA samples were purified and synthesized to cDNA. After cluster amplification was conducted through PCR, a sequencing library was sequenced with Illumina HiSeqTM2000. Raw sequences were filtered to remove the joint, undetected sequences and low-quality sequences. Transcriptional transcripts were constructed from the remaining sequence, and known non-coding RNAs and protein-encoding fragments were discarded. The new non-coded sequence and the known non-coding sequence were compared and statistically analyzed to screen the differentially expressed lncRNAs in the two-time-point tissue samples.

On the basis of the Poisson distribution model, we simultaneously calculated the differential expression multiples [log2(E18D/P2W)] of lncRNA in E18D and P2W samples in accordance with the gene expression (RPKM value) and examined the differences in *P*-value by controlling the false discovery rate (FDR) to determine the *P*-value of the domain. LncRNAs with | log2(E18D/P2W) | >4 and FDR < 0.01 were considered differentially expressed in E18D and P2W samples.

### RNA-FISH Assay

The mandible samples of embryonic (16, 18, and 20 days old) and postnatal (2 and 4 weeks old) C57 mice were collected and fixed in 4% paraformaldehyde for 48 h and then decalcified in EDTA. The decalcified specimens were paraffin embedded, and 5 mm serial sections were prepared for the experiments. The slides were hybridized with lnc-DMP1 and *Dmp1* probes overnight at 37°C. Afterward, the slides were washed twice with 2 × saline sodium citrate (SSC) and three times with deionized water formamide: 2 × SSC (50:50) for 20 min. DAPI solution was used to stain the cell nuclei. Fluorescence images were acquired through fluorescence microscopy.

### Overexpressing and RNAi of lnc-DMP1

For lnc-DMP1 overexpression, the full length of lnc-DMP1 was cloned into the lentivirus vector pCDH-CMV-MCS-EF1-copGFP (Gene, Shanghai, China). The shRNA sequence targeting lnc-DMP1 was cloned into the lentivirus vector psi-LVRU6MH (Gene) for the interference of the lnc-DMP1 expression. All of the vectors were transfected into MC3T3-E1 cells in accordance with the manufacturer’s instructions. The efficiency of transfection was confirmed through qRT-PCR. The cells were subjected to RNA extraction, Western blot assay and functional assays.

### Quantitative Real-Time PCR Assay (qRT-PCR)

The total RNA of mandible samples and cells was extracted with TRIzol reagent in accordance with the manufacturer’s instructions (Thermo Fisher Scientific) and reverse transcribed with a high-capcity cDNA reverse transcription kit (Vazyme, Nanjing, China). SYBR Green PCR master Mix (Vazyme) and primers (Table 1) were used for qRT-PCR. The qRT-PCR program was set at the following parameters: 95°C for 2 min, followed by 40 cycles at 95°C for 15 s and 60°C for 32 s. *GAPDH* was used as reference genes, and the relative gene expression level was calculated by using the comparative threshold (Ct) cycle method (2^–ΔΔ*Ct*^).

**TABLE 1:** Sequences of primers used in this study.

Primer name	Sequence (5′-3′)	Purpose
*Dmp1*-Q-F	GCTGGTATCAGGTCGGAAGAATC	qRT-PCR
*Dmp1*-Q-R	CCTGCTGTTGCTGTCAGTAAGC	qRT-PCR
lnc-DMP1-Q-F	TCAAGCAAGCTCACCAGACA	qRT-PCR
lnc-DMP1-Q-R	CCGTCAGCATGACAGTTCCA	qRT-PCR
*GADPH*-Q-F	GGCCTCCAAGGAGTAAGAAA	qRT-PCR
*GADPH*-Q-R	GCCCCTCCTGTTATTATGG	qRT-PCR
pCDH-lnc-DMP1-F	AAAATCTAGAAGGAAAAC AGAGCCCTGCTACTATG	lnc-DMP1 OE
pCDH-lnc-DMP1-R	AAAAGCGGCCGCACTGCC TATTTAATGAATGCGACC	lnc-DMP1 OE
sh-lnc-DMP1-F	AAAGAATTCAAAAATCCTGCCTC TTAGCAGCTCGAGAAA	lnc-DMP1 RNAi
sh-lnc-DMP1-R	TTTCTCGAGCTGCTAAGAGGC AGGATTTTTGAATTCTTT	lnc-DMP1 RNAi
PGL3-*pro:Dmp1*-F	CGGGTACCCTGCTCACTGATA GGCAAGCCTTC	Luciferase assay
PGL3-*pro:Dmp1*-R	CCGCTCGAGAGAAGGCTTGT CTGACAGTGCAG	Luciferase assay
*Dmp1*-p-F	GCAAAAGATATATATTTAGAAAG	ChIP-qRT-PCR
*Dmp1*-p-R	CATACACCCACACTTCCTCCAG	ChIP-qRT-PCR
*GAPDH*-p-F	CATGGGTGTGAACCATGAGA	ChIP-qRT-PCR
*GAPDH*-p-R	GTCTTCTGGGTGGCAGTGAT	ChIP-qRT-PCR

### Western Blot Assay

The cultured cells were extracted by RIPA (Radio Immunoprecipitation Assay) ordinary type reagent (Vazyme), which is a traditional cell tissue lysis buffer to gain proteins for western and IP experiments. Then, 30 μg of each protein was loaded to each lane, fractionated by 10% SDS-PAGE, transferred onto PVDF membranes and probed with the primary antibody of DMP1 and DSPP (Abcam, Cambridge, United Kingdom) as a target and GAPDH (CST, Boston, MA, United States) as an internal control at 4°C overnight. The secondary antibodies were added for 2 h. The blots were visualized through enhanced chemiluminescence and autoradiography.

### Induction of Odontoblast-Like Differentiation and Alizarin Red Staining

The lnc-DMP1 overexpression and RNAi stable cell lines were induced by osteoinductive factors (50 μg/mL ascorbic acid phosphate, 10 mM β-glycerol phosphate disodium salt and 10 nmol/L dexamethasone) for 28 days. After mineralization was induced, the medium was discarded. The cells were rinsed with PBS three times, fixed with 95% ethanol for 20 min and stained in alizarin red solution for 10 min. The calcification nodules formed in each group were compared.

### Luciferase Assay

The dual-luciferase reporter vector pGL3-Basic vector, which contains renilla luciferase with 35S promoter and firefly luciferase without driven promoter, was used to construct the pGL3-*pDmp1* reporter vector that including both renilla luciferase with 35S promoter and firefly luciferase driven by the *Dmp1* promoter (2000 bp before transcription start site). The plasmids of CMV empty vector, CMV-lnc-DMP1, U6-Sh empty vector and U6-Sh-lnc-DMP1 were co-transfected with pGL3-*pDmp1* plasmid into MC3T3-E1 cell. Lipofectamine 2000 was used. After 48 h of transfection, the cells were collected, and luciferase activity was measured by using a dual luciferase reporter assay kit (Promega, Madison, United States) in accordance with the manufacturer’s protocol. The firefly luciferase activities of the *Dmp1* promoter co-transfected with CMV vectors or U6-Sh vectors were firstly normalized to renilla luciferase activity of itself, then showed as a relative LUC activity to the empty vector control CMV-lnc-DMP1 or U6-Sh empty vector.

### ChIP

ChIP was conducted by using a ChIP kit (Qiagen, Düsseldorf, Germany) in accordance with the manufacturer’s instructions. In brief, the cells were incubated with formaldehyde, and the link between DNA and protein was built. Then, the cross-linked chromatin was sonicated into fragments. Anti-H3K27ac antibodies (Abcam) were used for the immunoprecipitation of the chromatin fragments, and IgG was used as the negative control. Subsequently, the precipitated chromatin DNA was measured through qRT-PCR.

### DNA Pull Down

*In vitro*, part of purified PCR product pDmp1 DNAs, which was 378 bp length of Dmp1 promoter before the transcriptional start site (TSS), were biotin-labeled with a Biotin DNA labeling mix (Roche, Basel, Switzerland). The Biotin-labeled and unbiotin-labeled pDmp1 DNAs were mixed with MC3T3-E1 cell protein extract in 500 μL incubation buffer (RIP buffer (100 mM NaCl, 10 mM Tris, 1 mM EDTA, 1 mM EGTA, PH7.6) with 0.2% TritonX 100 and protease inhibitor) and then incubated at 4°C overnight. Afterward, 50 μL of washed streptavidin agarose beads (Thermo Fisher Scientific) was added to each binding reaction and further incubated at RT for 1 h. The beads were washed briefly with wash buffer [RIP buffer (100 mM NaCl, 10 mM Tris, 1 mM EDTA, 1 mM EGTA, PH7.6) with 0.1% TritonX 100] five times and boiled in SDS buffer. The retrieved proteins were detected by Western blot.

### Primer Sequences

The primer sequences are shown in Table 1.

### Statistical Analysis

Data were analyzed with SPSS 20.0 and presented as mean ± SD. Differences between the two groups were assessed by using two-tailed Student’s *t*-test. Differences with a *P* < 0.05 were considered statistically significant (^∗^*P* < 0.05 and ^∗∗^*P* < 0.01).

## Results

### Expression Profile Analysis of lncRNAs in the Development of Mouse Mandibles

**FIGURE 1:**
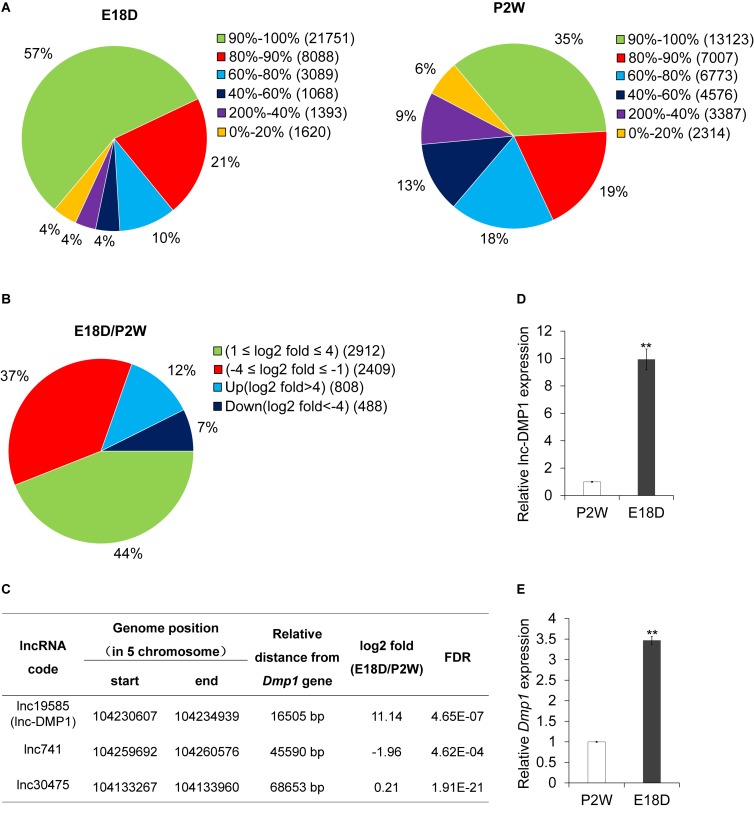
lnc-DMP1 is a novel lncRNA near the *Dmp1* gene region, differentially expressed in E18D and P2W samples. **(A)** The distribution of lncRNAs’ coverage in E18D and P2W samples from lncRNA-seq data. **(B)** The distribution of differentially expressed lncRNAs (| log2 fold change (E18D/P2W) | ≥ 1, FDR < 0.001) in E18D and P2W samples. **(C)** lncRNAs located within 100 kb of *Dmp1* (Chromosome 5:104202613-104214102). **(D)** and **(E)** qRT-PCR detection of lnc-DMP1 **(D)** and *Dmp1* **(E)** in E18D and P2W samples. Data were normalized to *glyceraldehyde-3-phosphate dehydrogenase* (*GAPDH*) expression, and P2W was set to a value of 1. **P < 0.01 by Student’s *t*-test. E18D, embryonic18-day-old mouse mandible; P2W, postnatal 2-week-old mouse mandible.

To examine the expression profiles of lncRNAs in the development of mouse mandibles, we performed lncRNA sequencing (lncRNA-seq) analysis. RNA was collected from the mouse mandibles of embryonic18-day-old (E18D) and postnatal 2-week-old (P2W) mice. A total of 38,566 lncRNAs were identified in the two samples (Figure 1A). Through stringent classification (| log2 fold change (E18D/P2W) | >4, FDR < 0.001), 808 and 408 lncRNAs were found to be up-regulated (log2 fold change (E18D/P2W) >4) and down-regulated (log2 fold change (E18D/P2W) < −4) (Figure 1B), respectively. These results suggested that lncRNAs might be involved in the regulation of mouse mandible development.

### lnc-DMP1 Differentially Expressed in the Development of Mouse Mandibles and Located Near the *Dmp1* Gene Region

To investigate lncRNAs that might modulate the transcriptional expression of *Dmp1* in *cis*, we searched lncRNAs located within 100 kb of the *Dmp1* gene region (Chromosome 5:104202613-104214102) from the lncRNA-seq data. We found that lnc19585 and lnc741 were located downstream of *Dmp1*, with distances of 16.7 and 45.6 kb, respectively, whereas lnc30475 was located upstream of *Dmp1*, with distance of 68.6 kb (Figure 1C). Amongst these three lncRNAs, lnc19585 was the nearest to *Dmp1* and the only one that was up-regulated in the E18D sample relative to the P2W sample. Thus, we renamed lnc19585 as lnc-DMP1.

To confirm the results of the lnc-DMP1 expression from lncRNA-seq, we also conducted qRT-qPCR to detect the expression levels of lnc-DMP1 and *Dmp1* in the mandibles derived from E18D and P2W mice. The results showed that the level of lnc-DMP1 was remarkably up-regulated in E18D compared with that in P2W (Figure 1D), and this expression pattern was consistent with that of *Dmp1* (Figure 1E). These results indicated that lnc-DMP1 was a novel lncRNA near the *Dmp1* gene region, which was differentially expressed in the development of mouse mandibles.

### Co-localization of lnc-DMP1 and *Dmp1* in the Development of Mouse Mandibles

**FIGURE 2:**
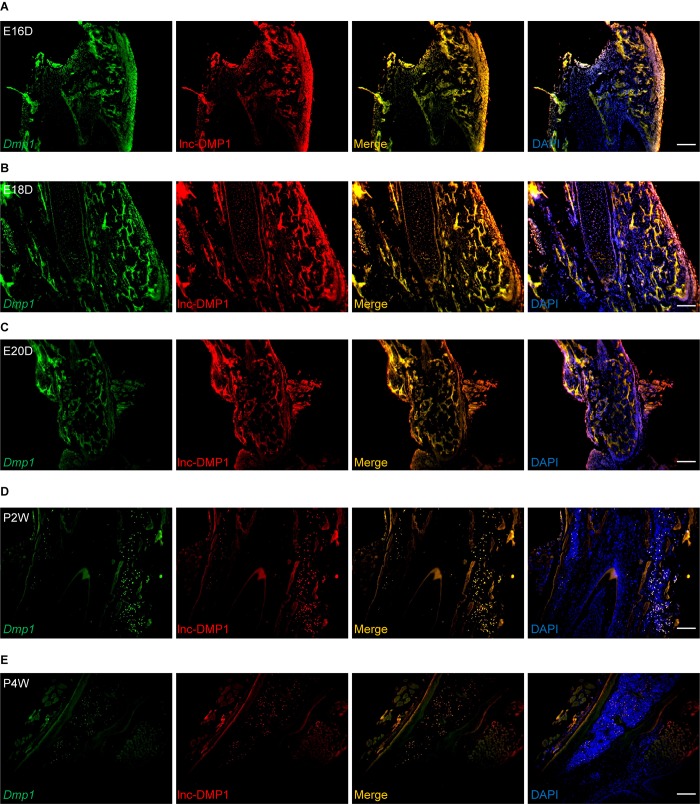
RNA-FISH assay of lnc-DMP1 and *Dmp1* in the development of mouse mandibles. Representative images of lnc-DMP1 (green) and *Dmp1* (red) expression in embryonic 16- **(A)**, 18- **(B)** and 20-day **(C)** mouse mandibles and in postnatal 2- **(D)** and 4-week **(E)** mouse mandibles. Yellow denoted the co-localization of lnc-DMP1 and *Dmp1*. Nuclei were stained with DAPI (blue). Scale bar represented 500 μm.

To test whether lnc-DMP1 was co-localized with *Dmp1*, we performed RNA-FISH to detect the expression of lnc-DMP1 and *Dmp1* in the mandibles derived from embryonic 16-, 18-, and 20-day-old mice and postnatal 2- and 4-week-old mice. In Figure 2, the expression levels of *Dmp1* (green fluorescence) and lnc-Dmp1 (red fluorescence) were highly co-localized (yellow fluorescence) in all of the developmental processes of mouse mandibles, and the co-localization was distributed in the region, including the nuclei (blue fluorescence) and the cytoplasm. We found that the expression levels of lnc-DMP1 and *Dmp1* decreased in the postnatal mouse mandibles (Figures 2D,E) compared with those in the embryonic mouse mandibles (Figures 2A–C). These data suggested that the expression of lnc-DMP1 was significantly correlated with that of *Dmp1* in different developmental processes of mouse mandibles.

### lnc-DMP1 Positively Regulates *Dmp1* Expression and Mineralization in MC3T3-E1 Cells

**FIGURE 3:**
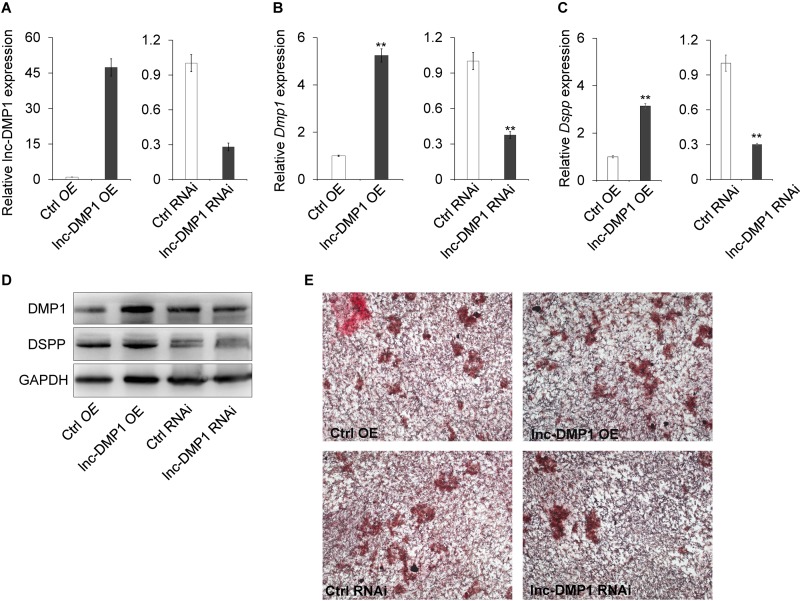
lnc-DMP1 controls mineralization by increasing the DMP1 expression in MC3T3-E1 cells. **(A)** The transformation efficiency of lnc-DMP1 OE and RNAi stable lines in MC3T3-E1 cells was determined through qRT-PCR. Data were normalised to *GAPDH* expression, and the empty vector control was set to a value of 1. **(B,C)** The alteration of the mRNA levels of *Dmp1* **(B)** and *Dspp* **(C)** in lnc-DMP1 OE and RNAi cells. **(D)** The alteration of the protein levels of DMP1 and DSPP in lnc-DMP1 OE and RNAi cells. **(E)** Mineralization was detected by subjecting lnc-DMP1 OE and RNAi cells to alizarin red staining. **P < 0.01 by Student’s *t*-test.

To test the role of lnc-DMP1 on the modulation of *Dmp1* expression, we selected MC3T3-E1 cells to construct stable OE and RNAi lines of lnc-DMP1, which were, respectively, transduced with the expression vectors of pCDH-CMV-lnc-DMP1 and psi-LVRU6MH-sh-lnc-DMP1, and the empty vectors were transduced as control lines (Figure 3A). The mRNA expression level of *Dmp1* was detected in stable lnc-DMP1 OE and RNAi lines, and qRT-PCR results showed that the *Dmp1* expression increased in the lnc-DMP1 OE line and decreased in the lnc-DMP1 RNAi line relative to the control lines (Figure 3B). *Dspp* was a downstream gene of *Dmp1*, whose expression was regulated by *Dmp1*. We also detected the mRNA expression level of *Dspp* in lnc-DMP1 OE and RNAi lines, and the results showed the same expression pattern of *Dspp* as *Dmp1* in these stable lines (Figure 3C). We further tested the alteration on the protein level through an immunoblot assay. The results showed that the protein levels of DMP1 and DSPP increased in the lnc-DMP1 OE line and decreased in the lnc-DMP1 RNAi line (Figure 3D). These data suggested that lnc-DMP1 acted as an upstream regulator promoted both mRNA and protein expression of DMP1.

Given that *Dmp1* plays an important role in the control of osteoblast cell mineralization (Toyosawa et al., 2001; Ling et al., 2005), we determined whether lnc-DMP1 participated in the skeletal mineralization of osteoblast cells. The results of the cell mineralization assays performed after the cells were treated with osteo-inductive factors for 28 days showed that more mineralized nodules were formed in the lnc-DMP1 OE line compared with those in the control lines, whereas the lnc-DMP1 RNAi line showed obviously inhibited mineralized nodules formation compared with that in the control lines (Figure 3E). These results suggested that lnc-DMP1 controlled mineralization by increasing the *Dmp1* expression in MC3T3-E1 cells.

### Roles of lnc-DMP1 in Modulating the Activity and H3K27ac Enrichment of *Dmp1* Promoter

**FIGURE 4:**
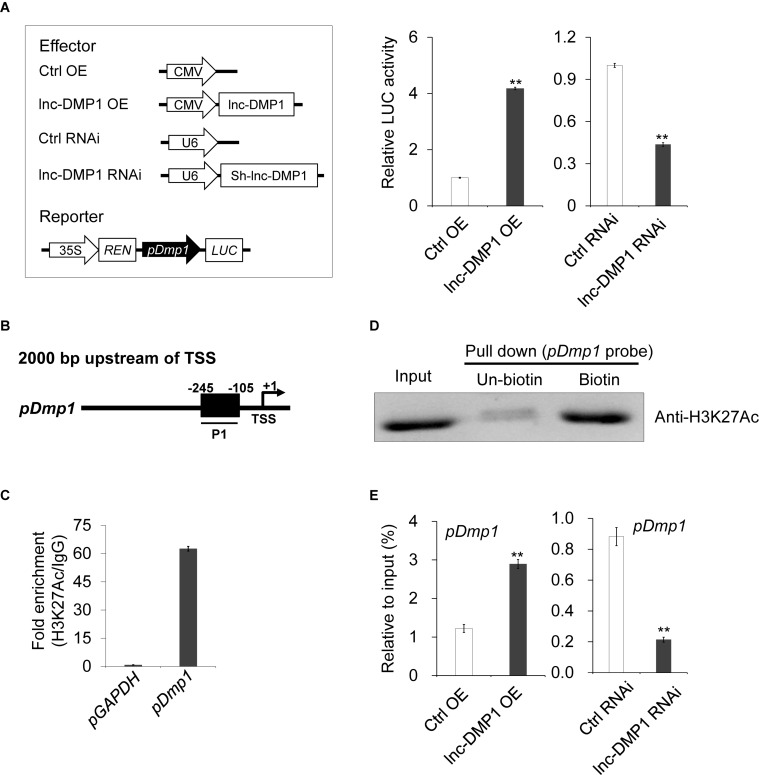
lnc-DMP1 induces the promoter activity by modulating the H3K27Ac enrichment of the *Dmp1* promoter region in MC3T3-E1 cells. **(A)** Luciferase (LUC) reporter assay: the *Dmp1* promoter region [2000 bp before transcription start site (TSS)] was cloned upstream of the firefly luciferase coding region. Their luciferase activities were tested in MC3T3-E1 co-transfected with CMV:Lnc-DMP1 (lnc-DMP1 OE), U6:sh-lnc-DMP1 (lnc-DMP1 RNAi) or empty vectors (Ctrl OE and Ctrl RNAi). The empty vector control was set to a value of 1. **(B)** Schematic of the potential H3K27Ac binding region in the promoter sequence of *Dmp1*. The numbers indicated the nucleotide positions relative to their transcription start site (TSS), which was shown as + 1. **(C)** qRT-PCR detection of the indicated DNAs retrieved by H3K27Ac-specific antibody compared with immunoglobulin G (IgG) in the CHIP assay within MC3T3-E1. **(D)** DNA pull down experiment with MC3T3-E1 extract. Specific bands were identified by immunoblotting H3K27Ac. **(E)** The alteration of the enrichment of H3K27Ac in the promoter region of *Dmp1* in lnc-DMP1 OE and RNAi cells. **P < 0.01 by Student’s *t*-test.

To test whether lnc-DMP1 functions in the transcriptional regulation of *Dmp1* expression, we made a construct expressing a luciferase reporter gene under the control of the *Dmp1* promoter. While the *pDmp1:LUC* reporter gene was co-transfected with CMV-lnc-DMP1 plasmid into MC3T3-E1 cells, the expression of luciferase significantly increased relative to that of the control. The co-transfected of U6-sh-lnc-DMP1 plasmid reduced the reporter gene expression (Figure 4A). These results suggested that lnc-DMP1 induced the promoter activity of *Dmp1*.

lncRNA can bind adaptor protein and target transferase to drive histone modification and active gene transcription (Marchese et al., 2017). According to the ENCODE (Encyclopedia of DNA Elements) project data, we could not find any histone modification information of *Dmp1* in mouse cells. Nonetheless, histone H3-lysine-27 acetylation (H3K27Ac) was enriched in the promoter region of *Dmp1* in human H1-hESC cells. Hence, we conducted a ChIP assay to detect the enrichment of H3K27Ac in the promoter of *Dmp1* in MC3T3-E1 cells. The results showed that the acetylation of H3K27 in the promoter of *Dmp1* was enriched (Figures 4B,C). The DNA pull down assay also confirmed the binding of *Dmp1* promoter to the H3K27Ac protein (Figure 4D).

Given that the enhanced H3K27Ac histone modification is usually an important agonist for transcriptional regulation, we hypothesized that lnc-DMP1 participated in the acetylation of H3K27 in the promoter of *Dmp1*. To confirm this hypothesis, we performed ChIP assay to detect the enrichment of H3K27Ac in the promoter of *Dmp1* in lnc-DMP1 OE and lnc-DMP1 RNAi stable lines. As shown in Figure 4E, the overexpression of lnc-DMP1 increased the enrichment of H3K27Ac of the *Dmp1* promoter, whereas the RNAi of lnc-DMP1 decreased the enrichment of H3K27Ac of the *Dmp1* promoter. These results suggested that lnc-DMP1 induced the promoter activity by modulating the H3K27Ac enrichment of *Dmp1* promoter.

## Discussion

lncRNAs play important regulatory roles during multiple biological processes and in various diseases (Wang et al., 2015; Ding et al., 2017). Histone modification through methylation and acetylation is the main regulatory mechanism by which lncRNAs regulate the expression of their neighbor protein-coding genes. lncRNAs modulate the expression of target genes by promoting the acetylation of H3K27 in the promoter regions of genes. *Dmp1* is highly expressed in bone and dentin tissues and plays a key role in mineralization, but the mechanism of its regulation needs to further investigated (Narayanan et al., 2002; Jacob et al., 2014; Wang et al., 2014). In this study, we attempted to investigate the potential regulatory pattern in lncRNAs and *Dmp1*.

lnc-DMP1, a novel lncRNA validated by the lncRNA sequence in our study, was near the *Dmp1* gene region and exhibited the most remarkable changes during mandible development. The RNA-FISH assay demonstrated that lnc-DMP1 and *Dmp1* were expressed in the same areas in the mandibles and co-localized in the cytoplasm and the nuclei. Moreover, the significant correlation between the expression levels of lnc-DMP1 and *Dmp1* at different stages of mandible development implied the presence of a regulatory mechanism between them. After constructing the stable lines of lnc-DMP1 overexpression and RNAi in osteoblast cell lineMC3T-E1, we found that overexpressing lnc-DMP1 promoted both mRNA and protein expression of DMP1 and skeletal mineralization in MC3T3-E1 cells. These results indicated that lnc-DMP1 positively regulated the expression of DMP1 to control the mineralization. The luciferase results implied that lnc-DMP1 induced the promoter activity of *Dmp1*. Given that the acetylation level of H3K27 was positively correlated with the transcriptional activity, the results of the ChIP assays confirmed our hypothesis that lnc-DMP1 enhanced *Dmp1* expression by enriching H3K27Ac in *Dmp1* promoter.

In summary, our study identified a novel lnc-DMP1 positively related to the *Dmp1* during the development of mouse mandibles. The lnc-DMP1 regulated *Dmp1* expression and mineralization by modulating the H3K27ac enrichment of *Dmp1* promoter in osteoblast cells. In our future studies, we will further investigate chromatin-modifying complexes, which interact with lnc-DMP1 to modulate *Dmp1* expression. Collectively, our findings will serve as a basis for exploring the mechanism of lncRNAs in the regulation of bone regeneration, including periodontal tissue reconstruction, and for tissue engineering and clinical practice.

## Data Availability Statement

The lnc-RNA sequencing data mentioned in our manuscript has been submitted to the SRA in NCBI, the SubmissionID is BioProject ID is PRJNA540617 and BioSample accessions are SAMN11549457 and SAMN11549458.

## Author Contributions

XX, YR, BL, YY, XK, and PZ performed the experiments and/or analyzed the data. HW designed the research. YR participated the design of some experiments. XX and HW wrote the manuscript. All co-authors corrected the manuscript.

## Conflict of Interest

The authors declare that the research was conducted in the absence of any commercial or financial relationships that could be construed as a potential conflict of interest.
